# Screening and personalizing nootropic drugs and cognitive modulator regimens *in silico*

**DOI:** 10.3389/fnsys.2015.00004

**Published:** 2015-02-06

**Authors:** Leslie C. Jellen, Alexander Aliper, Anton Buzdin, Alex Zhavoronkov

**Affiliations:** ^1^Department of Genetics, Genomics, and Informatics, University of Tennessee Health Science CenterMemphis, TN, USA; ^2^Aging Research, Insilico Medicine, Emerging Technology Center, Johns Hopkins University EasternBaltimore, MD, USA; ^3^Personalized Medicine, Pathway Pharmaceuticals LtdWan Chai, Hong Kong; ^4^Research, Biogerontology Research FoundationTruro, UK

**Keywords:** nootropic, cognitive enhancement, personalized medicine, drug repositioning, oncofinder, *in silico* medicine, signalome, signalome profiling

## Abstract

The go-to cognitive enhancers of today are those that are widely available rather than optimal for the user, including drugs typically prescribed for treatment of ADHD (e.g., methylphenidate) and sleep disturbances such as narcolepsy (modafinil). While highly effective in their intended therapeutic role, performance gains in healthy populations are modest at best and profoundly inconsistent across subgroups and individuals. We propose a method for *in silico* screening of possible novel cognitive enhancers followed by high-throughput *in vivo* and *in vitro* validation. The proposed method uses gene expression data to evaluate the the collection of activated or suppressed signaling pathways in tissues or neurons of the cognitively enhanced brain. An algorithm maps expression data onto signaling pathways and quantifies their individual activation strength. The collective pathways and their activation form what we term the signaling pathway cloud, a biological fingerprint of cognitive enhancement (or any other condition of interest). Drugs can then be screened and ranked based on their ability to minimize, mimic, or exaggerate pathway activation or suppression within that cloud. Using this approach, one may predict the efficacy of many drugs that may enhance various aspects of cognition before costly preclinical studies and clinical trials are undertaken.

## Introduction

The concept of cognitive enhancement is age-old, but despite modern advances in our understanding of the cellular and molecular bases of cognition, much remains unknown. Development of new cognitive enhancers with increased specificity, safety, and effectiveness must outpace increasing demand, particularly as off-label and illicit use of current drugs has become common in certain population subgroups (Maher, 2008) and as the aging segment of the population expands with accompanying increases in rates of cognitive decline and neurodegenerative disease (Wallace et al., 2011).

Cognition is complex and involves multiple domains, from learning and memory to attention, and cognitive enhancers can target one or more. At present, two of the most widely cited cognitive enhancers are the dopaminergic stimulants methylphenidate, prescribed for treatment of ADHD, and modafinil, prescribed for treatment of sleep disorders, such as narcolepsy. While both of these drugs can enhance memory and attention in healthy individuals (Elliott et al., 1997), their application as nootropics is secondary to their original, therapeutic purpose, and effects are disagreed upon and modest at best (Repantis et al., 2010).

A number of other potential nootropics have been developed for the treatment of cognitive deficits in aging and neurodegenerative or neuropsychiatric disease, from the FDA approved acetylcholinesterase inhibitors (e.g., donepezil) to those still under investigation: ampakines, nicotinic receptor agonists, glutamate receptor agonists, glycine inhibitors, and PDE inhibitors (for review, Wallace et al., 2011; Pieramico et al., 2014). Drugs that may play a more modulatory role target histamine, serotonin, glucocorticoid, and neuropeptide receptors and epigenetic mechanisms (Roesler and Schröder, 2011; Wallace et al., 2011). Many other drugs positioned as nootropic agents like piracetam and piracetam-like compounds are well-tolerated, but their effects and mechanisms of action are poorly understood and widely debated (Gouliaev and Senning, 1994; Gualtieri et al., 2002). While some nootropics have made it to clinical trial, others still await or have been withdrawn, and each has various drawbacks or only modest effects that are disease specific.

Development of novel nootropics is hampered by research, validation and regulatory challenges. The very definition of cognitive enhancement is difficult to pin down (Lynch et al., 2014). The road from lab to FDA approval is difficult, long, and costly. Pharmacological enhancement of healthy populations is fraught with ethical and philosophical pushback (Maslen et al., 2014). Enhancement in aging and neurodegenerative disease is less controversial, but perhaps more complex (Pieramico et al., 2014). Moreover, therapeutic effects often contradict those in healthy populations (Kimberg et al., 1997; Belmonte and Yurgelun-Todd, 2003; Beglinger et al., 2005; Gibbs and D’Esposito, 2005, 2006; Randall et al., 2005; Frank and O’Reilly, 2006; Finke et al., 2010; Esposito et al., 2013). The U-shaped curve effect, wherein treatment effects benefit low-baseline performers but impair high-performers, is a problem with at least the dopaminergic drugs (Gibbs and D’Esposito, 2006; Finke et al., 2010; Esposito et al., 2013). Even drugs FDA-approved for therapeutic use have issues with side effects, trade-offs (one process is enhanced while another is impaired), loss of authenticity (one’s true “self”), and large individual differences (Maslen et al., 2014). Long term effects are typically unknown. Finally, and most importantly, there is still much to be learned about the cellular and molecular basis for the various aspects of cognition.

For the field of neuroenhancement to advance, the benefit-to-risk ratio of pharmacological treatment must improve. This will require (a) a better understanding of the neurobiological basis of cognition; and (b) drugs with higher specificity, greater effectiveness, and fewer side effects. To this end we propose an expedited path to understanding the molecular basis of cognition and to subsequent drug discovery via a combination of advanced gene expression analysis, signaling pathway analysis, and *in silico* screening of potential nootropics. In our approach, current drugs will simply be the launching pad on the quest to find new, better performing nootropics.

## Signaling pathway activation profiles as drug targets for predicting nootropic effects *in silico*

Enhancing cognition is a challenge as it consists of various processes each influenced by many factors and each with a distinct neurobiological framework. While single-gene studies have been an effective first step in identifying individual elements necessary for learning and memory to occur (Lee and Silva, 2009), in moving forward, integration of many other factors and influences, including the relatively few signaling pathways involved, may be more beneficial than investigating each of many potential individual network elements.

Intracellular signaling pathways show promise for complex trait analysis. At the cellular level, any two physiological states can be distinguished by changes in a set of signaling pathways, each individually activated or repressed to some extent. This collection of disturbed pathways, termed the signaling pathway cloud, is a powerful and unique biological fingerprint (Zhavoronkov and Cantor, 2011; Makarev et al., 2014; Aliper et al., 2015).

Until recently, signaling pathway analysis was impeded by the inability to quantify individual signaling pathway activation strength (PAS), owing to the complexity of protein interactions within signaling pathways and lack of experimental data to determine the importance factor for each member of a given pathway. This problem was addressed, however, with the development of Oncofinder^1^, a biomathematical method that simplifies calculation of PAS and signaling pathway cloud disturbance (SPCD), as detailed below (Buzdin et al., 2014b). With the ability to calculate PAS, one can quantitatively characterize a biological condition by its associated signaling activation profile.

PAS analysis involves (1) mapping relevant pathways from the gene expression profiles for a given condition; (2) calculating individual PAS values; (3) constructing the signaling pathway cloud (net activated and repressed pathways); (4) high throughput *in silico* screening to predict and rate drugs that target these pathways, depending on the application; and (5) *in vitro* and *in vivo* validation.

The pair of conditions to be compared in signaling pathway cloud analysis is flexible. In the past, we have compared tumor biopsies to healthy tissue, revealing signaling pathway biomarkers that outperform those of single genes (Kuzmin et al., 2010; Mityaev et al., 2010; Zabolotneva et al., 2012a,b), and tissue from old vs. young patients, illustrating how this method may be useful for the screening of potential geroprotectors (Zhavoronkov and Cantor, 2011). Other applications include drug discovery and drug repurposing, as well as applications in manipulating cell differentiation.

**Figure 1:**
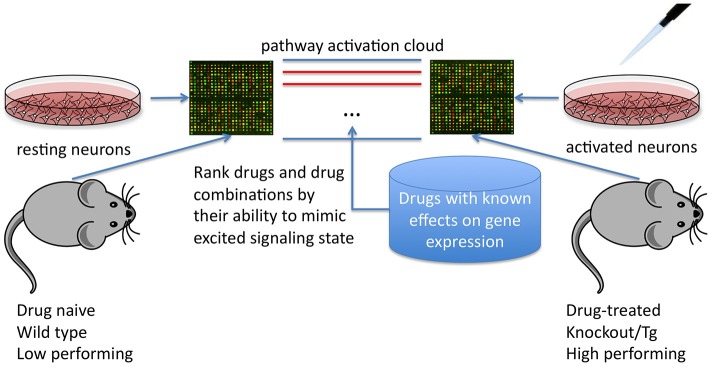
**Using differential signaling pathway activation profiles between cell/tissue culture or mice treated and untreated with known cognitive modulators or genetic models of cognitive enhancement to screen for potential nootropic compounds**.

Here, we suggest that these methods could also be applied to cognitive enhancement, first by defining what aspect of cognition is to be targeted, then by mapping signaling pathways altered in transcription profiles of the “enhanced brain” vs. control (e.g., in mouse models showing performance gains in the target cognitive domain, whether via genetic manipulation, selective breeding or inbreeding, or treatment with current nootropics) and finally by calculating disturbance of the associated signaling pathway cloud (Figure 1).

High throughput screening for drugs that activate or repress key pathways could then uncover drugs that replicate cloud disturbance in “enhanced” models and thus mimic or exaggerate the effects of the current drugs or genetic manipulations. These drugs could then be ranked and prioritized for validation of cognitive effects *in vivo* or *in vitro*.

These methods can be used for general screening of cognitive enhancers at present but in the future may also be used to develop personalized cognitive enhancement plans for individual patients.

The idea of using differential gene expression patterns for drug discovery is not new. The broad variability of as well as the error rates introduced by the microarray and next generation sequencing (NGS) equipment led to many early failures, impeded progress and diminished the potential of this approach. However, recent algorithmic solutions reducing complexity and smoothing variability of the gene expression mapped onto signaling pathways allow us to minimize errors and perform cross-platform analysis (Buzdin et al., 2014b). These solutions allow us to tap into legacy databases and compare hundreds of thousands of data sets in microarray and NGS repositories as well as utilize the drug effects on gene expression published in publically available databases.

## Step 1: identifying the pair of conditions

Enhanced cognition can refer to augmented function across one or several domains and can be demonstrated in a number of highly specific paradigms or tests, thus one must first specify the goals of enhancement and choose models/tissues that demonstrate enhancement in that respect.

Ethical considerations preclude the use of human studies for this application, thus animal models would be required. Fortunately, a number of animal models are highly useful in investigating mechanisms of cognition (Roesler and Schröder, 2011). Specific aspects of attention, learning, and memory can be reliably measured in a wide variety of behavioral paradigms. Many of these have been developed for use in mice, but rats, monkeys, zebrafish, and drosophila are also potential models. For the current approach, mouse models are promising, and there are several different options within this species for comparison of the “enhanced” vs. “non-enhanced” brain.

## Mutant vs. wild type

Mice are a classic model for learning and memory and a number of models involve mutations that induce cognitive enhancement, including knockout and transgenic mutations (Lee and Silva, 2009). These model systems have revealed many genes involved in learning and memory. Hypothetically, the human orthologs of these genes would also be involved in cognition. Mutations that enhance cognition are varied in mechanism, and include enhancing excitation, dampening inhibition, regulating gene expression, regulating translation, epigenetics, microRNA biogenesis, and extracellular molecules (Lee and Silva, 2009). Generally speaking, mutations that increase the activity of cAMP response element-binding protein (CREB), a basic leucine zipper transcription factor, enhance long-term potentiation (LTP) and both long and short term memory, while those that downregulate CREB activity impair LTP and memory (Lee and Silva, 2009; Suzuki et al., 2011; Kida and Serita, 2014). In their 2009 review, Lee and Silva reported that 26 of the 29 mouse models reviewed showed enhanced LTP. In several cases, LTP was enhanced, but cognition was either not improved or was actually impaired; thus, other signaling pathways and cellular processes are involved (Lee and Silva, 2009), underscoring the potential role for signaling pathway analysis in elucidating global effects on these mice and thereby enabling pharmacological replication.

## Strain vs. strain

An alternative to single gene mutation models is selective breeding or inbreeding to exploit “natural” genetic variation among mouse strains in cognitive performance. Strains or groups of strains that perform high on paradigms of learning and memory can be compared to other low-performing strains within a genetic reference panel. The BXD strains are a good example (Williams et al., 2003). This panel of recombinant inbred strains has been extensively phenotyped for behaviors related to learning and memory and drug treatment effects as well as profiled for basal gene expression, and the data are publicly available for rapid statistical analysis on genenetwork.org (Wang et al., 2003). In minutes, known phenotypic differences can be correlated to known gene expression levels (and thus signaling pathway activation), without the unwanted or exaggerated effects of knockout or transgenic manipulation.

## Drug-treated vs. drug-naïve

A third alternative for comparisons in the analysis would be *in vivo* or *in vitro* expression in organisms, tissues, or cells that have been pharmacologically treated with current nootropics vs. control. The effect of nootropics on gene expression in the brain can be large; in the case of methylphenidate treatment, over 2000 genes have been shown to be differentially expressed in the caudate putamen of mice (Adriani et al., 2006a,b). This comparison would enable screening of new nootropics that mimic current nootropics, but that are more effective, more or less specific in targeted cognitive domains, and/or have fewer side effects.

## Step 2: identifying target signaling pathways

Once a specific comparison is decided upon, a predicted set of relevant pathways can be assembled from the literature or mapped from gene expression data. Here, we reviewed the literature for mouse models of cognitive enhancement, in which overexpression, knockout, loss-of-function mutation, deletion, or RNA interference of a particular gene led to significant gains in cognitive performance as measured in at least one behavioral paradigm for measuring learning and/or memory. In 2009, Lee and Silva reviewed these models and compiled list of target genes and effects of mutation. We reduced this list to only 40 genes, for which human orthologs exist. Gene set enrichment analyses of these listed entries against Insilico Cloud Intelligence signaling pathway database (Buzdin et al., 2014a) were performed using Fisher’s exact test and the most enriched pathways are shown in Table 1. Results revealed several overrepresented signaling pathways, including IP3, IGF1R, and cAMP molecular signalization, potentially important for learning and memory in humans. Key activators/repressors of these pathways can therefore be used in further experimental assays together with the mathematical apparatus we provide in the following section.

**Table 1:** **Signaling pathways associated with cognitive enhancement in animal models**.

Pathway name	Overlap with gene list (%)	Odds ratio	***p*-value**
IP3 pathway (gene expression)	25.9	0.346	1.53*E*-08
IGF1R pathway (cell survival)	17.9	0.215	2.39*E*-07
cAMP pathway (axonal growth)	20.8	0.245	6.88*E*-06
IGF1R main pathway	6.6	0.073	2.09*E*-05
IP3 main pathway	5.2	0.058	0.000142525
IL-2 main pathway	4.4	0.044	0.002495586
Wnt main pathway	2.8	0.029	0.028947377
GPCR main pathway	2.5	0.025	0.033190669
cAMP main pathway	2.1	0.021	0.034326773

*Gene set enrichment analyses of the genes implicated in cognitive performance against Insilico Cloud Intelligence signaling pathway database performed using Fisher’s exact test and the most enriched pathways*.

Importantly, many of the genes and pathways identified during *in silico* screening of nootropics are also implicated in aging and longevity (Zhavoronkov and Cantor, 2011; Moskalev et al., 2014) thus suggesting that geroprotector drugs may act as nootropic agents and vice versa.

## Step 3: calculating signaling pathway cloud disturbance

The work we propose involves calculating signaling activation and methods are based on our prior work with cell signaling pathways (Kiyatkin et al., 2006; Kuzmina and Borisov, 2011; Borisov et al., 2014). In the past, calculation of signaling pathway activation has been avoided because of the lack of experimental data measuring the correlation between expression and activation at the protein level. In our observation, most signal transduction proteins are far from saturation even at the peak concentrations of the activated form, in comparison with total protein abundance. From this, we suggest all activator/repressor gene products/proteins have equal importance for pathway activation/downregulation. We then arrive at the following assessment: function for overall signal pathway cloud disturbance outcome (SPCD) is proportional to the following estimator function:

$$SPCD=\frac{\prod_{i=1}^{N}{\left[AGEL\right]}_{i}}{\prod_{j=1}^{M}{\left[RGEL\right]}_{j}}$$

Here, the multiplication is performed over all possible activator and repressor proteins in the pathway, and [*AGEL*]_*i*_ and [*RGEL*]_*j*_ are gene expression levels of an activator *i* and repressor *j*, respectively. To obtain an additive (not multiplicative) value, one can simply switch from using absolute values of expression levels to their logarithms, arriving at the *PAS* value for each pathway

$$PAS_{p}=\sum_{n}ARR_{np}\cdot BTIF_{n}\cdot \mathrm{lg}\left(ECR_{n}\right)$$

In the case of cognitive enhancement, to obtain the values of *enhanced-to-control ratio (ECR)*, one divides the expression levels for a gene *n* in the sample taken for the enhanced group by the same average value for the normalized control group. The discrete value of activator/repressor role (ARR) is the relative strength of the target activation or repression.

The information about the activator/repressor role of a particular gene product/protein may be obtained from the analysis of open-access or customized pathway databases and from the literature. The Boolean flag of *BTIF* (beyond tolerance interval flag) equals to zero when the ECR value lies within the tolerance limit, and to one when otherwise. We determined that the ECR lies beyond the tolerance limit if it satisfies simultaneously the two criteria. First, it should be either higher than 3/2 or lower than 2/3 of the corresponding gene expression level in normal group of samples, and, second, the expression level of a gene should differ by more than two standard deviations from the average expression level for the same gene in a control group of samples.

## Limitations of differential signaling pathway analysis-based drug screening methods

Acquiring gene expression data from the various regions of human brain during excitation is difficult and human data would have to be obtained from post mortem tissues or cultured cells or tissues. Gene expression data from mouse brain is easier to obtain, but further studies are required to evaluate the correlations between signaling pathway activation profiles in mice and humans. Tissue selection is also important, gene expression in one region or cell type may or may not be representative of other areas of the brain and may not capture all effects of a given condition. Also, gene expression alone may not provide the complete picture of the state of the tissue, and while the pathway activation analysis approach may be used to analyze differences in genomic DNA (Spirin et al., 2014) there may be epigenetic regulation of cognitive states that may require other analytical methods to be performed. Aside from tissue selection, the complexity of cognition presents other challenge. The proposed approach evaluates the various drugs and drug combinations that mimic or enhance the effects of already known nootropics or genetic manipulations; however, no current nootropic agents optimally enhance specific aspects of cognitive function without side effects, and even genetic models of cognitive enhancement can produce unintended impairments (Lee and Silva, 2009). In this paper, we have referred to the “enhanced” brain vs. control in describing the comparisons one would use to perform signaling pathway analysis. However, “enhanced” is a simplified, general term that does not specify effects on the various cognitive domains, and defining enhancement remains an important issue in developing new nootropics (Lynch et al., 2014).

## Conclusion

Cognitive enhancement is in demand, whether in healthy populations or those with cognitive deficits, but current pharmacological enhancers offer only modest benefits. Testing of new cognitive enhancers is costly and time consuming. Even predicting the nootropic candidates out of the hundreds of thousands of drugs and drug combinations remains a major challenge. A screening process that would predict the efficacy of novel cognitive enhancers that may outperform current options would save time and money, both of which are limiting as the aging segment of the population explodes over the upcoming decades, with increasing rates of cognitive decline and neurodegenerative disease. Here we propose a method for *in silico* screening and ranking of drugs and other factors that act on signaling pathways involved in cognition. Predicting their efficacy would involve calculating their potential to maximize the difference in signaling pathway activation between cells or tissues of cognitively enhanced animal models, including mutant “smart” vs. wild type mice, high-performing vs. low-performing strains of mice, or drug-treated vs. drug naïve mice. This would be followed by *in vitro* and *in vivo* validation leading to a short list of promising components.

## Conflict of interest statement

Alex Zhavoronkov, Alex Aliper and Anton Buzdin are affiliated with the commercial companies searching for cognitive modulators and nootropics. The intent of this paper is to provide insight into the methods explored by these companies and shortlist the pathways implicated in cognitive performance.

## Footnotes

^1^www.oncofinder.com

## References

[B1] Adriani W. Leo D. Greco D. Rea M. di Porzio U. Laviola G.. (2006a). Methylphenidate administration to adolescent rats determines plastic changes in reward-related behavior and striatal gene expression. Neuropsychopharmacology 31, 1946–1956. 10.1038/sj.npp.1300962 16319916

[B2] Adriani W. Leo D. Guarino M. Natoli A. Di Consiglio E. De Angelis G.. (2006b). Short-term effects of adolescent methylphenidate exposure on brain striatal gene expression and sexual/endocrine parameters in male rats. Ann. N Y Acad. Sci. 1074, 52–57. 10.1196/annals.1369.005 17105903

[B3] Aliper A. Csoka A. B. Buzdin A. Jetka T. Roumiantsev S. Moskalev A. (2015). Signaling pathway activation drift during aging: Hutchinson-Gilford progeria syndrome fibroblasts are comparable to normal middle-age and old-age cells. Aging (Albany NY) [Epub ahead of print]. 10.18632/aging.100717 PMC4350323 25587796

[B4] Beglinger L. J. Tangphao-Daniels O. Kareken D. A. Zhang L. Mohs R. Siemers E. R. (2005). Neuropsychological test performance in healthy elderly volunteers before and after donepezil administration: a randomized, controlled study. J. Clin. Psychopharmacol. 25, 159–165. 10.1097/01.jcp.0000155822.51962.b4 15738747

[B5] Belmonte M. K. Yurgelun-Todd D. A. (2003). Functional anatomy of impaired selective attention and compensatory processing in autism. Brain Res. Cogn. Brain Res. 17, 651–664. 10.1016/s0926-6410(03)00189-7 14561452

[B6] Borisov N. M. Terekhanova N. V. Aliper A. M. Venkova L. S. Smirnov P. Y. Roumiantsev S.. (2014). Signaling pathway activation profiles make better markers of cancer than expression of individual genes. Oncotarget 5, 10198–10205. 25415353 10.18632/oncotarget.2548 PMC4259415

[B7] Buzdin A. A. Zhavoronkov A. A. Korzinkin M. B. Roumiantsev S. A. Aliper A. M. Venkova L. S. (2014a). The OncoFinder algorithm for minimizing the errors introduced by the high-throughput methods of transcriptome analysis. Front. Mol. Biosci. 1:8 10.3389/fmolb.2014.00008 PMC4428387 25988149

[B8] Buzdin A. A. Zhavoronkov A. A. Korzinkin M. B. Vankova L. S. Zenin A. A. Smimov P. Y.. (2014b). Oncofinder, a new method for the analysis of intracellular signaling pathway activation using transcriptomic data. Front. Genet. 5:55. 10.3389/fgene.2014.00055 24723936 PMC3971199

[B9] Elliott R. Sahakian B. J. Matthews K. Bannerjea A. Rimmer J. Robbins T. W. (1997). Effects of methylphenidate on spatial working memory and planning in healthy young adults. Psychopharmacology (Berl) 131, 196–206. 10.1007/s002130050284 9201809

[B10] Esposito Pieramico, V. Ferretti A. Macchia A. Tommasi M. Saggino A. Ciavardelli D.. (2013). Acute effects of modafinil on brain resting state networks in young healthy subjects. PLoS One 8:e69224. 10.1371/journal.pone.0069224 23935959 PMC3723829

[B12] Finke K. Dodds C. M. Bublak P. Regenthal R. Baumann F. Manly T.. (2010). Effects of modafinil and methylphenidate on visual attention capacity: a TVA-based study. Psychopharmacology (Berl) 210, 317–329. 10.1007/s00213-010-1823-x 20352415

[B11] Frank M. J. O’Reilly R. C. (2006). A mechanistic account of striatal dopamine function in human cognition: psychopharmacological studies with cabergoline and haloperidol. Behav. Neurosci. 120, 497–517. 10.1037/0735-7044.120.3.497 16768602

[B13] Gibbs S. E. D’Esposito M. (2005). Individual capacity differences predict working memory performance and prefrontal activity following dopamine receptor stimulation. Cogn. Affect Behav. Neurosci. 5, 212–221. 10.3758/cabn.5.2.212 16180627

[B14] Gibbs S. E. D’Esposito M. (2006). A functional magnetic resonance imaging study of the effects of pergolide, a dopamine receptor agonist, on component processes of working memory. Neuroscience 139, 359–371. 10.1016/j.neuroscience.2005.11.055 16458442

[B15] Gouliaev A. H. Senning A. (1994). Piracetam and other structurally related nootropics. Brain Res. Rev. 19, 180–222. 10.1016/0165-0173(94)90011-6 8061686

[B16] Gualtieri F. Manetti D. Romanelli M. Ghelardini C. (2002). Design and study of piracetam-like nootropics, controversial members of the problematic class of cognition-enhancing drugs. Curr. Pharm. Des. 8, 125–138. 10.2174/1381612023396582 11812254

[B40] Kida S. Serita T. (2014). Functional roles of CREB as a positive regulator in the formation and enhancement of memory. Brain Res. Bull. 105, 17–24. 10.1016/j.brainresbull.2014.04.011 24811207

[B17] Kimberg D. Y. D’Esposito M. Farah M. J. (1997). Effects of bromocriptine on human subjects depend on working memory capacity. Neuroreport 8, 3581–3585. 10.1097/00001756-199711100-00032 9427330

[B18] Kiyatkin A. Aksamitiene E. Markevich N. I. Borisov N. M. Hoek J. B. Kholodenko B. N. (2006). Scaffolding protein Grb2-associated binder 1 sustains epidermal growth factor-induced mitogenic and survival signaling by multiple positive feedback loops. J. Biol. Chem. 281, 19925–19938. 10.1074/jbc.m600482200 16687399 PMC2312093

[B19] Kuzmin D. Gogvadze E. Kholodenko R. Grzela D. P. Mityaev M. Vinogradova T.. (2010). Novel strong tissue specific promoter for gene expression in human germ cells. BMC Biotechnol. 10:58. 10.1186/1472-6750-10-58 20716342 PMC2929213

[B20] Kuzmina N. B. Borisov N. M. (2011). Handling complex rule-based models of mitogenic cell signaling (on the example of ERK activation upon EGF Stimulation). Int. Proc. Chem. Biol. Environ. Eng. 5, 67–82.

[B21] Lee Y. S. Silva A. J. (2009). The molecular and cellular biology of enhanced cognition. Nat. Rev. Neurosc. 10, 126–140. 10.1038/nrn2572 19153576 PMC2664745

[B22] Lynch G. Cox C. D. Gall C. M. (2014). Pharmacological enhancement of memory or cognition in normal subjects. Front. Syst. Neurosci. 8:90. 10.3389/fnsys.2014.00090 24904313 PMC4033242

[B23] Maher B. (2008). Poll results: look who’s doping. Nature 452, 674–675. 10.1038/452674a 18401370

[B24] Makarev E. Cantor C. Zhavoronkov A. Buzdin A. Aliper A. Csoka A. B. (2014). Pathway activation profiling reveals new insights into age-related macular degeneration and provides avenues for therapeutic interventions. Aging (Albany NY) 6, 1064–1075. 25543336 10.18632/aging.100711 PMC4298366

[B25] Maslen H. Faulmuller N. Savulescu J. (2014). Pharmacological cognitive enhancement—how neuroscientific research could advance ethical debate. Front. Syst. Neurosci. 8:107. 10.3389/fnsys.2014.00107 24999320 PMC4052735

[B26] Mityaev M. V. Kopantzev E. P. Buzdin A. A. Vinogradova T. V. Sverdlov E. D. (2010). Enhancer element potentially involved in human survivin gene promoter regulation in lung cancer cell lines. Biochemistry (Mosc) 75, 182–191. 10.1134/s0006297910020082 20367605

[B27] Moskalev A. A. Aliper A. M. Smit-McBride Z. Buzdin A. Zhavoronkov A. (2014). Genetics and epigenetics of aging and longevity. Cell Cycle 13, 1063–1077. 10.4161/cc.28433 24603410 PMC4013158

[B28] Pieramico V. Esposito R. Cesinaro S. Frazzini V. Sensi S. (2014). Effects of non-pharmacological or pharmacological interventions on cognition and brain plasticity of aging individuals. Front. Syst. Neurosci. 8:153. 10.3389/fnsys.2014.00153 25228860 PMC4151335

[B29] Randall D. C. Shneerson J. M. File S. E. (2005). Cognitive effects of modafinil in student volunteers may depend on IQ. Pharmacol. Biochem. Behav. 82, 133–139. 10.1016/j.pbb.2005.07.019 16140369

[B30] Repantis D. Schlattmann P. Laisney O. Heuser I. (2010). Modafinil and methylphenidate for neuroenhancement in healthy individuals: a systematic review. Pharmacol. Res. 62, 187–206. 10.1016/j.phrs.2010.04.002 20416377

[B31] Roesler R. Schröder N. (2011). Cognitive enhancers: focus on modulatory signaling influencing memory consolidation. Pharmacol. Biochem. Behav. 99, 155–163. 10.1016/j.pbb.2010.12.028 21236291

[B32] Spirin P. V. Lebedev T. D. Orlova N. N. Gornostaeva A. S. Prokofjeva M. M. Nikitenko N. A.. (2014). Silencing AML1-ETO gene expression leads to simultaneous activation of both pro-apoptotic and proliferation signaling. Leukemia 28, 2222–2228. 10.1038/leu.2014.130 24727677

[B33] Suzuki A. Fukushima H. Mukawa T. Toyoda H. Wu L. J. Zhao M. G.. (2011). Upregulation of CREB-mediated transcription enhances both short- and long-term memory. J. Neurosci. 31, 8786–8802. 10.1523/jneurosci.3257-10.2011 21677163 PMC6622960

[B34] Wallace T. L. Ballard T. M. Pouzet B. Riedel W. J. Wettstein J. G. (2011). Drug targets for cognitive enhancement in neuropsychiatric disorders. Pharmacol. Biochem. Behav. 99, 130–145. 10.1016/j.pbb.2011.03.022 21463652

[B35] Wang J. Williams R. W. Manly K. F. (2003). WebQTL: web-based complex trait analysis. Neuroinformatics 1, 299–308. 10.1385/ni:1:4:299 15043217

[B36] Williams R. W. Gu J. Qi S. Lu L. (2003). The genetic structure of recombinant inbred mice: high-resolution consensus maps for complex trait analysis. Genome Biol. 2:RESEARCH0046. 10.1186/gb-2001-2-11-research0046 11737945 PMC59991

[B37] Zabolotneva A. A. Bantysh O. Suntsova M. V. Efimova N. Malakhova G. V. Schumann G. G.. (2012a). Transcriptional regulation of human-specific SVAF(1) retrotransposons by cis-regulatory MAST2 sequences. Gene 505, 128–136. 10.1016/j.gene.2012.05.016 22609064

[B38] Zabolotneva A. A. Zhavoronkov A. Garazha A. V. Roumiantsev S. A. Buzdin A. A. (2012b). Characteristic patterns of microRNA expression in human bladder cancer. Front. Genet. 3:310. 10.3389/fgene.2012.00310 23316212 PMC3539722

[B39] Zhavoronkov A. Cantor C. R. (2011). Methods for structuring scientific knowledge from many areas related to aging research. PLoS One 6:e22597. 10.1371/journal.pone.0022597 21799912 PMC3142169

